# Evaluating Epidemiological Risk by Using Open Contact Tracing Data: Correlational Study

**DOI:** 10.2196/28947

**Published:** 2021-08-02

**Authors:** Stefano Piotto, Luigi Di Biasi, Francesco Marrafino, Simona Concilio

**Affiliations:** 1 Department of Pharmacy University of Salerno Fisciano Italy; 2 Bionam Research Center for Biomaterials University of Salerno Fisciano Italy; 3 Department of Computer Sciences University of Salerno Fisciano Italy

**Keywords:** SARS-CoV-2, COVID-19, contact tracing, Bluetooth Low Energy, transmission dynamics, infection spread, mobile apps, mHealth, digital apps, mobile phone

## Abstract

**Background:**

During the 2020s, there has been extensive debate about the possibility of using contact tracing (CT) to contain the SARS-CoV-2 pandemic, and concerns have been raised about data security and privacy. Little has been said about the effectiveness of CT. In this paper, we present a real data analysis of a CT experiment that was conducted in Italy for 8 months and involved more than 100,000 CT app users.

**Objective:**

We aimed to discuss the technical and health aspects of using a centralized approach. We also aimed to show the correlation between the acquired contact data and the number of SARS-CoV-2–positive cases. Finally, we aimed to analyze CT data to define population behaviors and show the potential applications of real CT data.

**Methods:**

We collected, analyzed, and evaluated CT data on the duration, persistence, and frequency of contacts over several months of observation. A statistical test was conducted to determine whether there was a correlation between indices of behavior that were calculated from the data and the number of new SARS-CoV-2 infections in the population (new SARS-CoV-2–positive cases).

**Results:**

We found evidence of a correlation between a weighted measure of contacts and the number of new SARS-CoV-2–positive cases (Pearson coefficient=0.86), thereby paving the road to better and more accurate data analyses and spread predictions.

**Conclusions:**

Our data have been used to determine the most relevant epidemiological parameters and can be used to develop an agent-based system for simulating the effects of restrictions and vaccinations. Further, we demonstrated our system's ability to identify the physical locations where the probability of infection is the highest. All the data we collected are available to the scientific community for further analysis.

## Introduction

In China, during December 2019, SARS-CoV-2 was identified as a novel beta coronavirus. At the time of writing this paper (December 2020), SARS-CoV-2 has caused almost 60 million confirmed human infections worldwide and more than 1 million deaths since its discovery [1,2]. The disease caused by SARS-CoV-2 is called COVID-19, and the disease was declared a global pandemic on March 11, 2020 [3]. Containment measures are the first and most crucial step for rapidly halting an outbreak that could otherwise become an epidemic or even turn into a pandemic, such as the COVID-19 outbreak [4]. Notable examples of disease epidemics with a high occurrence of superspreading events (SSEs) are the SARS-CoV (severe acute respiratory syndrome coronavirus; 2002-2003) and MERS-CoV (Middle East respiratory syndrome coronavirus; since 2013) epidemics [5-9]. The basic reproduction number (R_0_) is a key measure of transmissibility. It is defined as the number of infected contacts that 1 infected individual generates on average during their infectious period. An R_0_ value of >1 means that a virus will continue its propagation among susceptible hosts. In contrast, an R_0_ of <1 means that it is certain that epidemic spread will stop [10,11]. The SARS-CoV and MERS-CoV have an R_0_ of around 3 [12]. For SARS-CoV-2, the estimated R_0_ ranges between 2 and 3 [9,13]. However, it is unknown as to what extent SSEs are involved in the spread of SARS-CoV-2 infection.

Lockdown was the most widespread pandemic containment response, and it was introduced at different levels by most affected countries. As already predicted by mathematical models [14] and proven by trends that were updated at the time of writing this paper, the contagion's spread resumed rapidly when lockdown countermeasures were lifted. Rapid and automatic contact tracing (CT) is an essential intervention for contagion containment [15-19]; however, user localization poses a privacy risk and reduces compliance rates [20]. According to the World Health Organization, CT involves the following three steps: the identification of a contact (identifying those that a confirmed positive patient had contact with based on the transmission modalities of the pathogen of interest), the listing of contacts (keeping a record of individuals who possibly had contact with infected patients and informing these individuals), and contact follow-up [21]. CT has a dual purpose—treating people who have possibly been exposed to infectious diseases and stopping the transmission chain to contain an epidemic. Due to the prevalence of smartphones, CT has the potential to become a powerful intervention; the vast majority of smartphone users carry their smartphone devices with them throughout the day, and smartphones can generate detailed GPS location information. However, due to the availability of users’ location data, there is growing concern about the infringement of an individual's right to privacy. An alternative is using other contact monitoring technologies that are based on proximity assessments rather than those based on location information [22]. It is important to note that this study does not constitute an endorsement or rejection of CT based on potential data security risks or privacy limitations. This study intends to assess whether and to what extent the acquisition of contact data helps with assessing the spread of SARS-CoV-2.

Technologies such as Bluetooth Low Energy allow for the evaluation of the distance between users without locating them and thus help with addressing the privacy issue. The number of CT apps that have been introduced since the beginning of the SARS-CoV-2 pandemic is considerable [23,24] and reflects governments' interest in automating the tracing of people who have had recent contact with individuals who tested positive for COVID-19. An app that uses a centralized approach was developed by the academic spin-off company of the University of Salerno—SoftMining (SM). The app [25] was supported by government agencies such as the Campania Region and was validated by more than 120,000 users; the app had peaks of more than 15,000 active daily users.

CT is a fundamental intervention for acquiring population data, which show how different population groups can behave differently. Such behaviors result in different risks of infection among group members. In Multimedia Appendix 1, we describe how CT data were acquired via the Bluetooth Low Energy technology of the SM-COVID-19 app and how data were clustered to obtain different mobility and behavior groups. In this paper, we discuss how we used Italian National Institute of Health data on contagion trends in Italy [26] to estimate a more precise number of SARS-CoV-2–positive cases that was less influenced by the number of tests performed on the population. In addition, we show the link between the acquired CT data and the number of new SARS-CoV-2–positive cases. This allowed us to define an epidemiological risk function that was based on the number of, frequency of, and distance between contacts. The risk function expresses the probability that an individual will become ill as a function of their age within a given period of time. This study aims to evaluate whether the use of CT can support the containment of an epidemic. The data acquired from CT were analyzed and correlated with data on the progression of SARS-CoV-2 infection.

This study was not conducted for commercial purposes; it was conducted for the purposes of academic research and aims to make CT data available to the scientific community for future research.

## Methods

### CT Data Acquisition

During the CT phase, the SM-COVID-19 app analyzed the environment and, at regular intervals, sent data on the duration of a contact and the instantaneous and average distances (over the time) of a contact to the server. App users could voluntarily decide to share location data as well. If they did, the server also received latitude, longitude, precision, and smartphone provider data. We provide the full description of the data acquisition procedures in Multimedia Appendices 1 and 2. The developed technologies allowed for high precision in distance calculations (less than 0.5 m under optimal conditions and after device calibration) and were implemented via the SM-COVID-19 app, which is available on Android and iOS smartphones (via TestFlight; Apple Inc). Daily data were anonymized and saved for further use, as described in Multimedia Appendix 3, in accordance with the General Data Protection Regulation. Anonymity was also guaranteed when the GPS localization function was enabled, as data were stored randomly in the database; the database did not present an individual user's location in a precise way. The app only used random 128-bit proximity IDs, and only the user's device kept track of the device IDs. The app’s functions were conducted and maintained with a back-end server, on which arbitrary identifiers were stored. Users could not be identified directly with app data, as only the app's random identifiers were stored on the server.

### Social Mobility Analysis

The data set obtained from the SM-COVID-19 app in the period of April to November 2020 was analyzed. The data set's structure is described in Multimedia Appendix 2. Reported data from August 1 to August 30, 2020, were obtained to analyze mobility data from a period when no lockdown measures were in place. Such data are useful for tracking movements in real situations. We removed users with less than 15 days of activity from our analysis to exclude users who may have deactivated the app. The cleaned data set was clustered. Before the clustering process, the t-distributed stochastic neighbor embedding machine learning algorithm was applied to the data set to reduce its dimensionality to 2. The clustering was carried out by using the Ward linkage method. This method allows the user to select the number of clusters arbitrarily. We analyzed the distribution of data for different numbers of clusters (2-10 clusters); the optimal distribution was obtained with 5 clusters. The average number of daily contacts and the SDs for the clusters are reported in Table 1. SDs were high, since every cluster had many users with 0-contact days among those with low- and high-contact days. As shown in Table 1, the population was divided into clusters of approximately the same size. However, cluster 5 was larger and included users who had a larger number of contacts. This cluster accounted for the population with the highest number of contacts and included users with the highest number of contacts and the highest mobility.

**Table 1:** The cluster data of active users for the period of August 1 to August 30, 2020.

Cluster number	Number of daily contacts based on Bluetooth Low Energy technology, mean (SD)^a^	Percentage of active users
1	23.40 (38.55)	14
2	12.05 (22.62)	19
3	41.95 (75.79)	17
4	69.91 (103.76)	20
5	121.48 (145.05)	30

^a^The average number of daily contacts for each cluster and SDs were calculated based on all cluster data (ie, from days 1 to 30).

### Data Availability

All data can be made available upon request from the authors or the SM-COVID-19 team [27].

## Results

### Statistical Analysis and Estimates of the Real Number of SARS-CoV-2–Positive Cases

**Figure 1:**
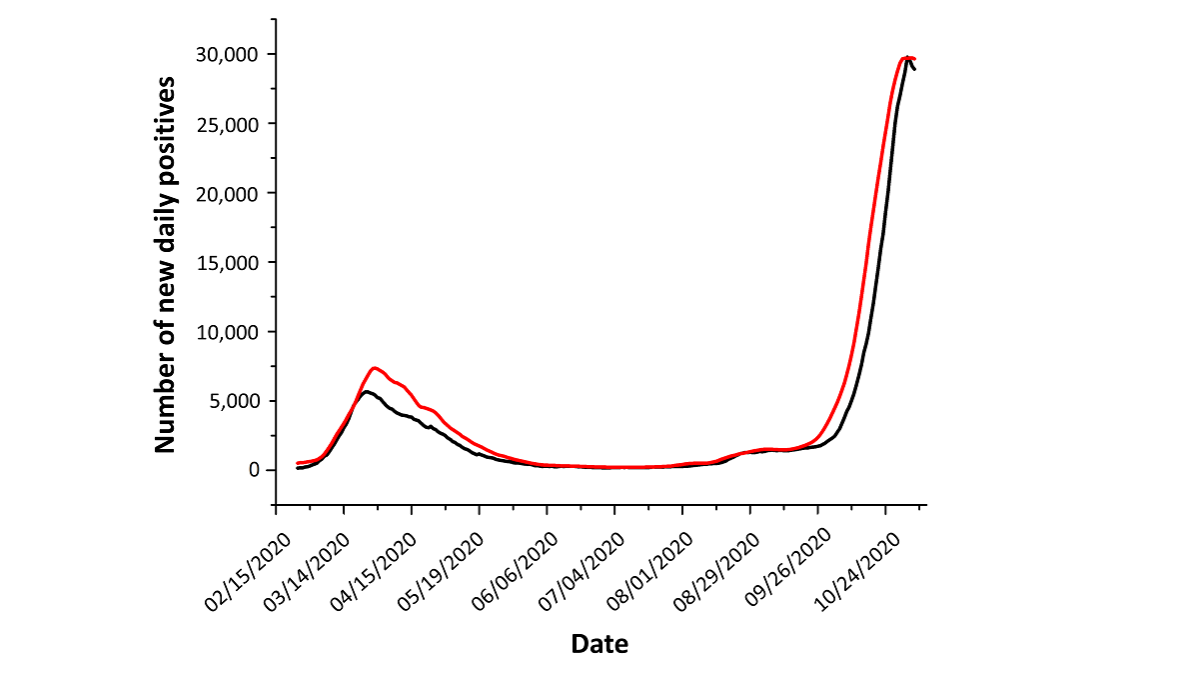
Comparison of the official number of daily new SARS-CoV-2–positive cases reported by the ISS (black line) and the estimated number of daily new SARS-CoV-2–positive cases (red line). The difference was higher during the initial phases of the pandemic. ISS: Istituto Superiore di Sanità (Italian Superior Institute of Health).

For our statistical analysis, we relied on official data on the daily SARS-CoV-2–related trends in Italy, which were released by the Italian National Institute of Health and aggregated by the Department of Civil Protection of the Presidency of the Council of Ministers [17]. We estimated the possible number of real infections that may have occurred during the epidemic in Italy. We obtained the daily number of newly performed tests based on the total number of tests performed. This was calculated by using equation 1 in Multimedia Appendix 4. The method for estimating the number of new daily SARS-CoV-2–positive cases is detailed in Multimedia Appendix 4. We performed data smoothing via sliding-window averaging to reduce each day's variability, which was the result of the cumulative regional data's intrinsic variability. The SARS-CoV-2–related trends over a given period were roughly linear; there were no sudden peaks. Additionally, the averaging process performed allowed us to smoothen the curves, which were in line with these trends. Equation 2 in Multimedia Appendix 4 was used to define the ratio between the number of daily tests and the number of daily reported SARS-CoV-2–positive cases. The estimated number of new SARS-CoV-2–positive cases (EP_[k]_) for each day was calculated with equation 3 in Multimedia Appendix 4. With our method, we estimated a correction for the number of real SARS-CoV-2–positive cases that occurred during the pandemic period. We observed that around 224,000 cases were not diagnosed, and of these cases, nearly around 81,000 were missed in the period of March to May 2020. The difference between the official number of cases and the estimated number of new SARS-CoV-2–positive cases is shown in Figure 1.

### Correlation Between CT Data and Contagion Trends

The correct number of daily SARS-CoV-2–positive cases was calculated to perform correlation analyses with the data obtained from CT. The data distributed by the ISS, due to how the data were structured, showed considerable fluctuations based on the number of tests performed. It was also possible to observe a weekly trend in the number of SARS-CoV-2–positive cases recorded due to the reduced number of tests performed during weekends. Such data therefore presented fluctuations that could alter the analysis. Data smoothing via sliding-window averaging also provided an additional element for alleviating the issue with fluctuations.

We then examined whether the contact index (CI) and the alpha index (α) correlated with the number of daily new SARS-CoV-2–positive cases. These two parameters are indices of effective contacts and account for the distance between two users who come into contact with each other and the contact's duration. These parameters and the related equations are described in detail in Multimedia Appendix 5 [4,28,29]. These parameters were necessary, since not all of the contacts recorded by the app involved people who could effectively transmit the virus. CI_k_ is a value that indicates a user’s risk of infection on day k based on the number of effective contacts that the user had on the same day. CI_k_ was calculated with equation 4 in Multimedia Appendix 5 [4,28,29]. α_k_ is a risk index, and it is based on data from the previous k−14 days (excluding day k). α_k_ reflects a user's behavior. The optimization of these parameters will be the subject of future studies.

**Figure 2:**
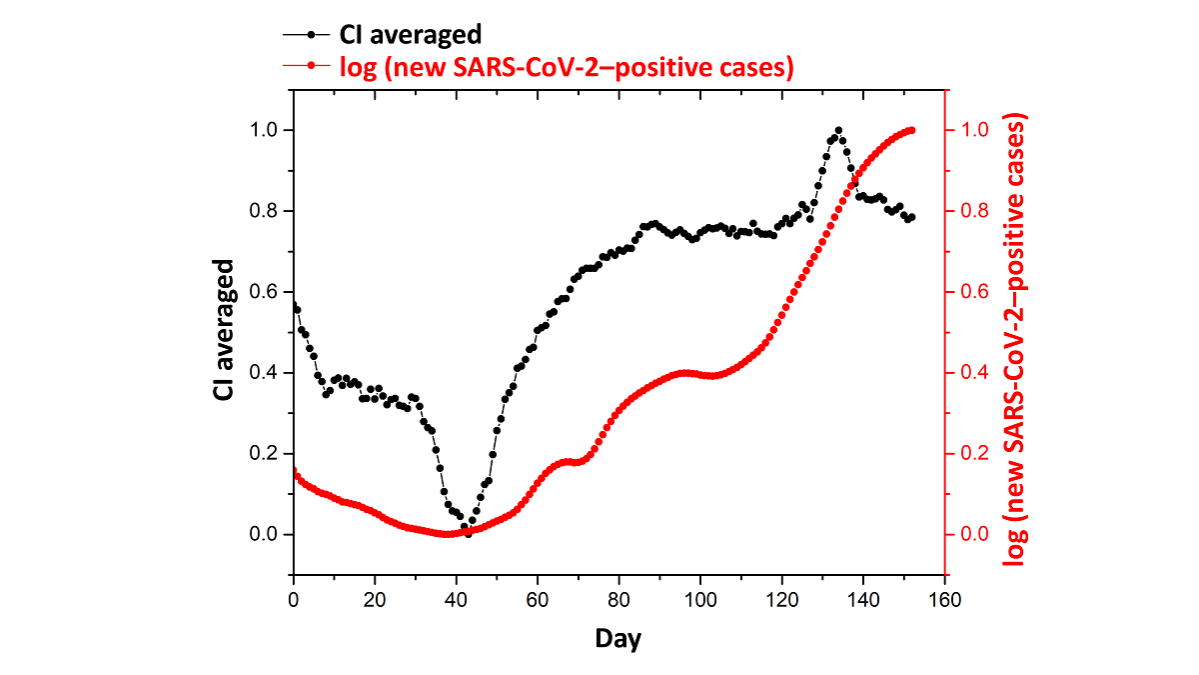
Temporal evolution of the CI values (black line) and the logarithm of the number of new SARS-CoV-2–positive cases (red line) during a 160-day period. CI: contact index.

**Figure 3:**
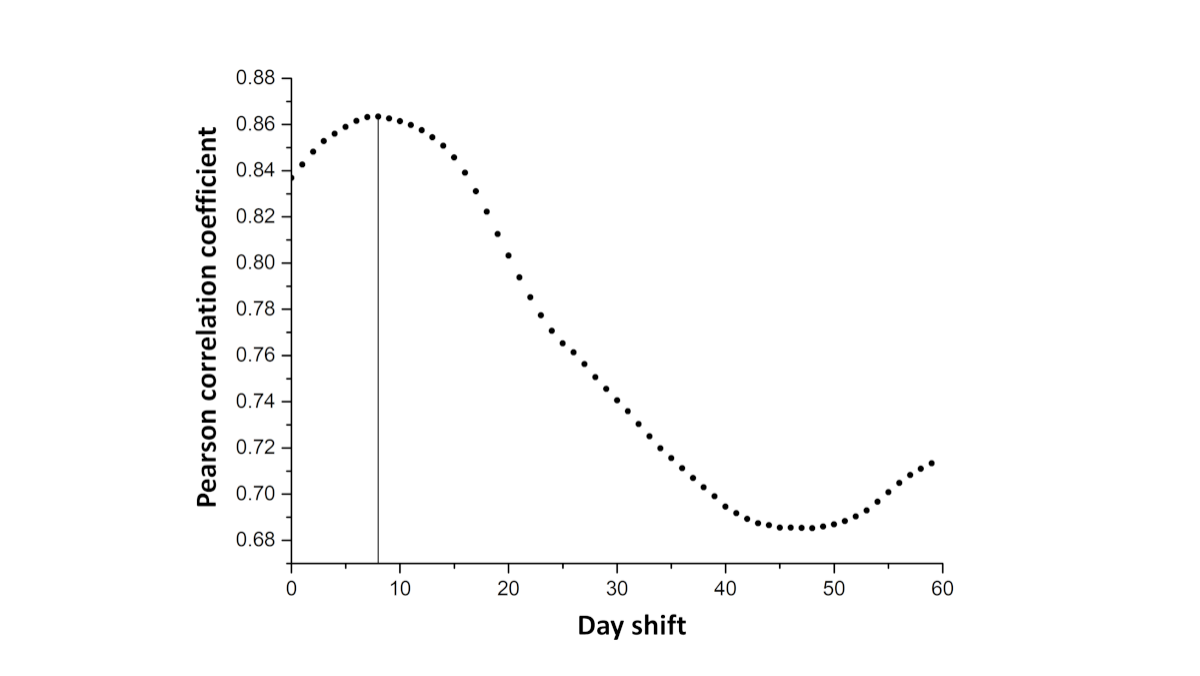
Pearson correlation analysis between CIk and the number of new SARS-CoV-2–positive cases with a time shift of 0 days to 60 days for the period of June 1 to October 31, 2020. The highest correlation value was observed at k+7 days. CI: contact index.

The SM-COVID-19 data set lists the CI and α values for each day and every user. Therefore, to evaluate daily trends, we calculated the total CI and α values for each day (k) by summing each individual users' values. As such, it was possible to evaluate the trends for CI and α values and exclude users who deactivated the app for a given period. The values were smoothed by using a sliding window of 7 days. In Figure 2, we show the temporal evolution of CI values over 160 days. For visualization, in Figure 2, we report the logarithm of the number of new SARS-CoV-2–positive cases. There is an evident, rough correlation between the CI and the number of new SARS-CoV-2–positive cases. For each CI_k_ and α_k_ value, we calculated the Pearson correlation coefficient based on the estimated number of SARS-CoV-2–positive cases to assess how the number of contacts varied before and after a confirmation of COVID-19 positivity. It was very interesting to note that the correlation coefficient for CI_k_ reached its maximum at k+7 days. The high correlations observed in the subsequent days correlated with SARS-CoV-2 incubation times, and COVID-19 positivity occurred in the days following an effective contact. The α_k_ value reached its maximum at k+5 days. The differences between the α and CI values’ correlation coefficients (ie, their correlation with the number of new SARS-CoV-2–positive cases) were attributable to the different calculation methods that were used for the two parameters, as the α value accounts for the risk of infection in the 14 days before day k. The correlation between CI values and the number of new SARS-CoV-2–positive cases is shown in Figure 3. We reported the correlation data that corresponded to the period of June to October 2020 because of the high availability of more consistent CT data. This correlation was also monitored for the previous studied period (March to May 2020) to confirm that the obtained values were not the result of artifacts or autocorrelations.

## Discussion

The analysis of the collected data allowed us to determine the aspects of CT that are essential for the evaluation of the progression of the SARS-CoV-2 pandemic. These essential aspects were identified via the estimation of the real number of new SARS-CoV-2–positive cases and the correlation of the number and frequency of contacts with the probability of infection.

### Estimation of the Total Number of People Who Tested Positive for SARS-CoV-2

At the beginning of the pandemic in Italy, during the period of March to May 2020, the substantial underestimation of the total number of people who tested positive for SARS-CoV-2 in Italy was a likely scenario. This was undoubtedly due to the reduced number of tests that were performed during the first phase of the SARS-CoV-2 pandemic and the lack of an adequate response for tracing infections. One method for estimating a realistic number of SARS-CoV-2–positive cases is to use the ratio between the number of tests carried out and the number of SARS-CoV-2–positive cases detected every day. We chose this ratio because as the number of tests carried out increases, this number eventually plateaus. These data are collected throughout the country and are therefore subject to regional and local variability. It has been assumed that the ratio between the number of positive cases and the number of tests performed varies slowly over time in the absence of hospitalization problems. This ratio has been used to estimate the actual number of SARS-CoV-2–positive cases, which is always greater than or equal to the official number of cases. As shown in Figure 1, the difference between the official number of daily new SARS-CoV-2–positive cases and the estimated number of cases was higher during the initial phases of the pandemic (ie, during the period of March to May 2020). During this period, according to our analysis, at least 81,000 patients with SARS-CoV-2 infection were not diagnosed with COVID-19. As already mentioned, calculating the real number of new SARS-CoV-2–positive cases was necessary because the data provided by the Istituto Superiore di Sanità (Italian Superior Institute of Health) varied according to the number of tests performed each day. In the initial stages of the pandemic, the number of tests was remarkably low due to the lack of adequate diagnostic tools.

### Ethical and Practical Issues of CT Apps

CT apps have generated much discussion, particularly discussions regarding privacy and such apps’ susceptibility to attacks. Considerations of data security and possible privacy violations are certainly essential elements and have resulted in the creation of numerous solutions that have been adopted at the national level. This paper does not aim not to take a position on the security and privacy of CT apps, although the developers of SM-COVID-19 have considered these aspects. Rather, we are concerned with assessing whether CT apps, that is, those that can be developed based on currently available technology, can impact communities' health. Several apps have been adopted at a national level by multiple countries. However, during our research, we did not find any information on the availability of data collected by these apps. CT data provide useful information on various aspects of the SARS-CoV-2 pandemic (eg, the pandemic course) and the behavior and mobility of app users, thereby allowing researchers to map the frequency of contacts and identify high-risk areas. Our CT data set allowed us to analyze data and identify different classes of behavior among the population.

The SM-COVID-19 app uses a centralized model [23,24]. However, despite using a centralized model, users' privacy is completely protected via anonymization, as per the General Data Protection Regulation. The advantage of using a centralized model is that data stored on the server can be anonymized via aggregation and used by public authorities as a source of important aggregate information about the number of contacts in the population, the app's effectiveness in tracing and alerting contacts, and the aggregate number of people who could potentially develop symptoms. Unlike a decentralized model, a centralized model provides access to CT data, thereby making these data available for analysis and the improvement of epidemiological models. As already stated by Ferretti et al [19], the control of the SARS-CoV-2 epidemic via manual CT is impossible, as CT introduces a time lag resulting from the need to notify individuals about having contact with infected individuals. Such lag exacerbates the spread the infection, which is already remarkable given the infectivity of SARS-CoV-2 and the high percentage of transmission by presymptomatic individuals. The use of this app model, in which individuals are immediately notified about having contact with people who tested positive for SARS-CoV-2, would be sufficient for stopping the epidemic if the app is used by an adequate number of people [30] and would provide valuable data for creating accurate and valid predictive and epidemiological models. The choice of using a centralized model allows for the reconstruction of the chains of contagion transmission and the rapid propagation of risk indices (calculated with mathematical models)—operations that are difficult to implement when tracing data are only kept on devices.

By using data from August 2020, during which no lockdown measures or restrictions on mobility were in place and only partial restrictions were placed on gatherings, it was possible to identify 5 different behavior classes (or mobility classes). Table 1 shows the data from the clustering process. The five groups had approximately the same population size except for cluster 5, which had the largest number of people and included individuals with the highest mobility. The high amount of deviation in cluster 5 shows how users in this class alternated between experiencing days with 0 contacts (ie, no mobility; eg, days when they could be working from home) and experiencing days with a very high number of contacts (eg, due to a commute or due to work involving contact with the public). From these clusters, it is impossible to define the reasons behind a given number of contacts, but this is irrelevant as long as similar behaviors are present among the users belonging to a certain cluster. However, this clustering process provided interesting insights; it showed that there are classes of people with very low mobility (eg, older people) and classes of people with high mobility who experience a high number of contacts (eg, working in a hospital, supermarket, etc). This information can be even more useful when using a localized approach, such as using GPS data, as such data would help with providing more appropriate definitions for categories. The contacts registered by the app allowed us to trace the frequency of contacts and the trend in the number of contacts for a given period, a single user, a cluster, or the whole data set.

### Correlation Between CT and the Total Number of New SARS-CoV-2–Positive Cases

CT data correlated with the growth in the number of new SARS-CoV-2–positive cases, and the highest correlation was observed 5 to 7 days after day k. This observation is in line with the hypothesis that an increase in the number of contacts is linked to an increased risk of infection. The most interesting element of the correlation is the time gap. The differences in the correlation values were probably related to the incubation period of SARS-CoV-2. Consequently, a contact that occurs on day k will not result in COVID-19 positivity on day k but on day k+n. This time gap is in line with the estimated incubation time for SARS-CoV-2 [4,28], and our analysis shows the effectiveness of using CT data to predict the number of new SARS-CoV-2–positive cases. This high correlation means that CT data can be used to develop new and more accurate epidemiological models and predictive tools.

Although a distributed approach that involves the use of a central advertising server makes it possible to alert individuals in direct contacts (the first contact between a newly infected individual and another person) about an eventual infection, flooding operations are necessary on CT networks to warn individuals about contacts of level 2 or higher. The decentralized model provides only 1 degree of separation from a CT app user who tested positive for COVID-19 (user A). To obtain data on a longer chain of contacts, which would have a decreasing risk gradient, it would be necessary for user B (a user in user A’s contact chain) to publish their identifier so that user C (a user who had contact with user B but not with user A) is alerted. This could prove particularly dangerous when an asymptomatic or low-symptomatic individual who has not been tested for SARS-CoV-2 infection could infect another person and even cause another person’s death. [31] In such a situation, decentralized CT would fail. On the other hand, the centralized model allows for the instant tracing of all contacts, regardless of the degree of separation. This would result in the more effective containment of the contagion, since all individuals in a contact chain that are deemed to be at risk for infection would be notified immediately about the danger. In this model, voluntary data input by individuals involved in first-degree contacts for informing those involved in second-degree contacts would not be required whenever the former was notified about having contact with a person who tested positive for COVID-19. Similar conclusions were reached by Aleta et al [30], who proved the effectiveness of using an automatic and extensive CT system to contain the spread of SARS-CoV-2 when lockdown measures are lifted. The work of Aleta et al [30] confirmed the usefulness of CT data collected from the population and provided an excellent basis for improving predictions and reducing the social and economic impact of SARS-CoV-2 prior to the effective vaccination of the entire population. At the time of writing this paper, we did not find any other available data sets with real CT data.

### Geolocalization

**Figure 4:**
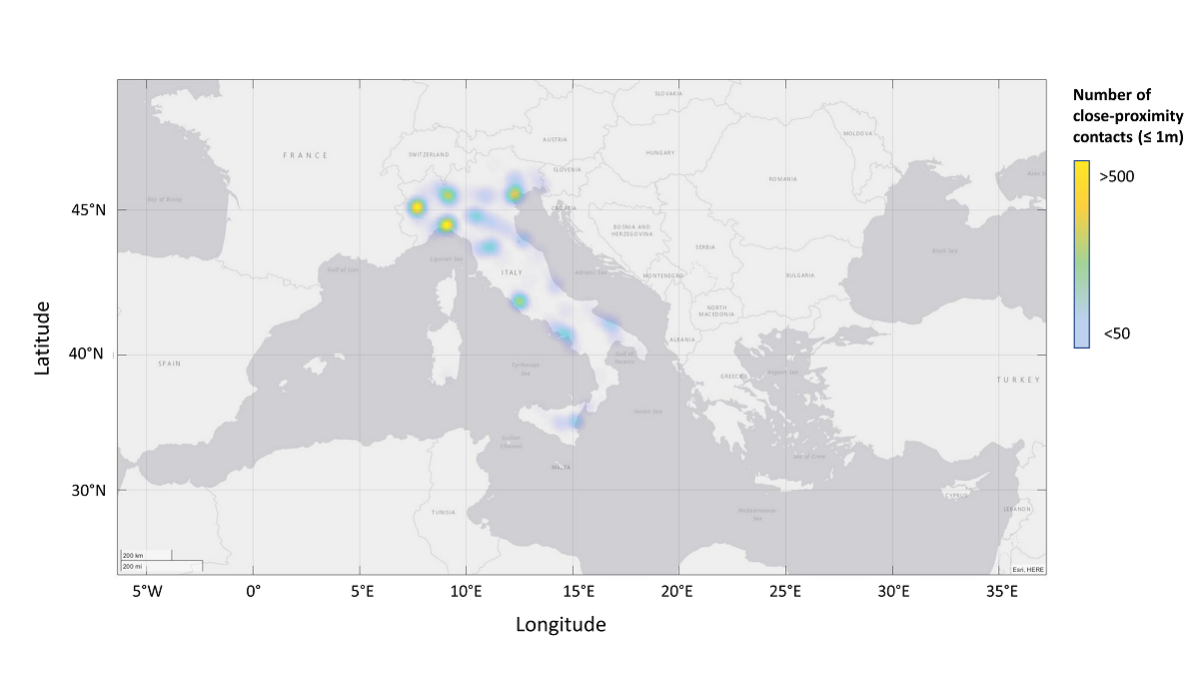
A map showing contact tracing app users’ GPS locations on September 10, 2020. These data were used for the tests.

**Figure 5:**
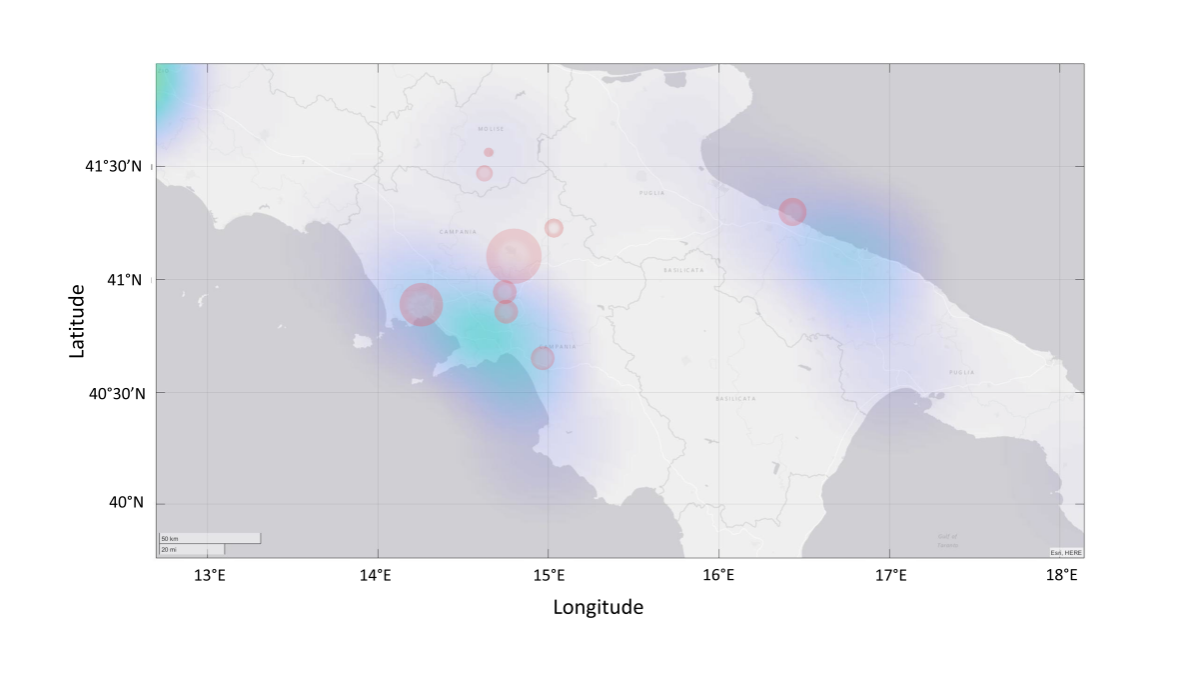
GPS test results. Green areas indicate locations that had a low risk of SARS-CoV-2 infection. Red areas indicate locations that had a high risk of infection. The red areas correspond to locations where SARS-CoV-2 outbreaks happened during the lockdown period.

CT data can be beneficial for evaluating SARS-CoV-2 propagation data. The data set that was made available by the app is particularly interesting because, due to its structure, it can be used as the basis for tracing SSEs. SSEs are generally defined as outbreaks in which a small number of individuals infect a large number of secondary individuals (ie, well-above the expected average number of individuals) [32]. The CT data that allowed us to define behavioral clusters for the population can also help with determining the SARS-CoV-2 pandemic’s potential for generating SSEs. Although lower than those of the SARS-CoV and MERS-CoV pandemics, the SARS-CoV-2 pandemic’s potential for generating SSEs is significant. In the absence of interventions such as social distancing, this potential would be even more significant. When developing disease control measures, people should focus on the rapid CT and quarantining of infected individuals and policies for physical distancing or targeted shutdowns to prevent the occurrence of SSEs. Having the ability to predict a pandemic’s potential for generating SSEs would be vital in preventing outbreaks, and it would considerably reduce a contagion’s overall R_0_ value. The use of GPS data that are made anonymous with an appropriate protocol would enable researchers to use a rapid localized approach to significantly reducing the risk of contagion spread in certain areas and act in a targeted and localized manner. This type of information can prove very useful for planning the possible containment of a contagion in defined areas. The tests we performed that used GPS data showed the potential of this approach. For these tests, CT data that were acquired during the lockdown period (April 14 to May 3, 2020) from SM-COVID-19 users who had explicitly activated GPS tracing and whose GPS coordinates included the Campania Region were used (Figure 4). The simulations showed that a higher number of alerts were generated in locations that corresponded to the outbreaks that occurred during the lockdown (Figure 5). This type of voluntarily provided information can be a handy tool for confining and preventing contagion spread.

### Conclusions

The high correlation between CT data and the number of recorded SARS-COV-2–positive cases (with a delay of 5-7 days) was remarkable. The number of registered contacts and the number of new SARS-COV-2–positive cases showed the same weekly trend fluctuations, which not only depended on the number of tests but also on the different mobility abilities of people. Moreover, there was a time lag between the two factors, and this was the result of the incubation time of SARS-CoV-2. This time lag can be used to estimate the real incubation time of SARS-CoV-2. Further, this correlation can be extremely useful for defining and predicting infection trends and can be used to improve predictive models that only use health authorities' data. Regardless of the effectiveness of CT, the collected data provided a powerful tool for improving predictive and epidemiological models and could be integrated into different types of analyses to improve the accuracy and efficiency of predictions based on real data.

This study lays the foundation for our upcoming papers. In future papers, we will show how CT data were implemented in a CT simulator to turn it into a real data-based contagion spread simulator, which provided us with data on the mobility of the different clusters that were defined in this study. The agents' mobility data will be used to determine the risk of infection, identify epidemiological parameters, and simulate the spread of SARS-CoV-2 in different contexts. The SM-COVID-19 data set is open and free for use by the scientific community. This paper does not represent a policy pronouncement, as this would not be a scientific objective. We believe that our study may prompt informed discussions of the possible risks and likely benefits of our approach to using CT data. For these reasons, all collected data are available for further analysis.

## Appendix

Multimedia Appendix 1 Acquisition of SM-COVID-19 app data on contacts.

Multimedia Appendix 2 Open data format.

Multimedia Appendix 3 Dumping and anonymization.

Multimedia Appendix 4 Statistical analysis and estimates of the real number of SARS-CoV-2–positive cases.

Multimedia Appendix 5 Contact index and alpha values.

## Abbreviations

CI: contact index
CT: contact tracing
MERS-COV: Middle East respiratory syndrome coronavirus
SARS-CoV: severe acute respiratory syndrome coronavirus
SM: SoftMining
SSE: superspreading event
