# Supplementary material for: Evaluating Epidemiological Risk by Using Open Contact Tracing Data: Correlational Study
Source: J Med Internet Res. 2021 Aug 2;23(8):e28947. doi: 10.2196/28947 (PMC8330631; doi:10.2196/28947)
Supplement: Multimedia Appendix 1 [file jmir_v23i8e28947_app1.docx]

## **Multimedia Appendix 1**

## **SM-Covid-19 app contacts acquisition**

During execution, the SM-Covid-19 app (APP) generates a unique 128-bit random ID (Proximity ID, PID) at regular intervals and transmits it to the environment using BLE technology. The APP also generates one single and immutable random ID (Running Instance ID, UUID), related to the app instance, that never changes during the APP lifecycle. UUID is never transmitted via BLE. The generated PID are subject to a remote Claim procedure to ensure that they have not been used in the past. The remote Claim service guarantees two properties:

- Atomicity: if two simultaneous calls to the Claim function contain the same UUID, one of the two is canceled.
- Non-repeatability: a UUID is accepted by the Claim phase and is authorized IF and ONLY IF it has not been previously acknowledged.

If the PID is declared unique, it is authorized to be transmitted, and the APP receives a temporary authentication token. The token will allow access to the other network services during the lifetime of the random ID. Each device keeps track locally of all the PIDs it has managed to register. Claim service can build a table containing the pairs (UUID, PIDs) to mitigate man in the middle and replay attacks. SM-Covid-19 runs data cleaning routines start every time the root user starts a data dump routine. The procedure is schematized in Figure 1.

*Figure SEQ Figure \* ARABIC 1: Registration protocol of a new UUID.*


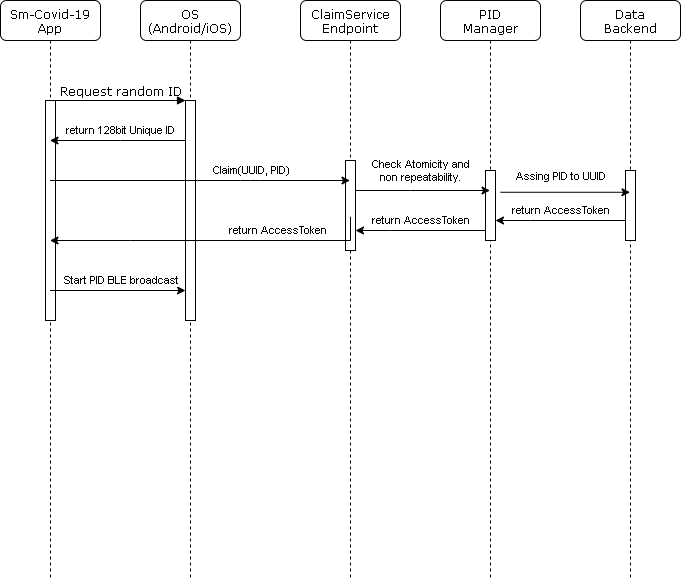


Figure 1

If the Claim procedure fails, the device is forced to generate a new temporary ID to use the network's services (Figure 2).

*Figure SEQ Figure \* ARABIC 2: Attempt of registration of an existing UUID.*


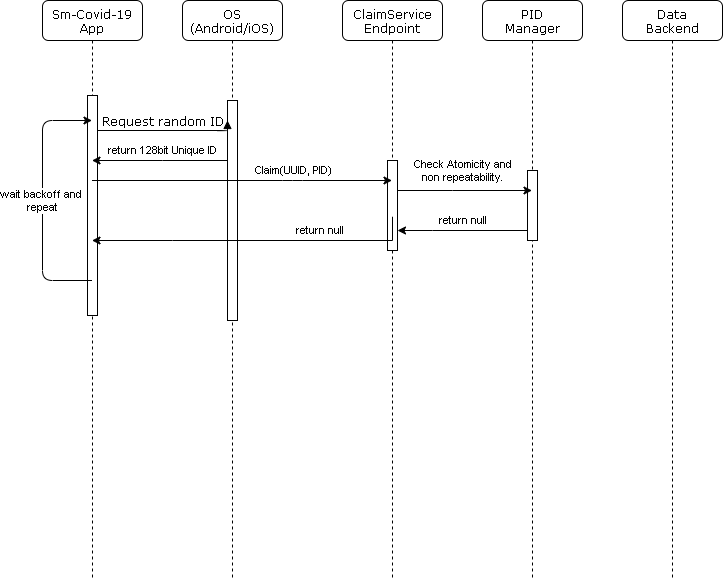


Figure 2

The contact tracing phase involves analyzing the environment searching for standard beacons (EddystoneUID, iBeacon, or AltBeacon). When a beacon is detected by a device (for example, received by APP1), the APP checks the beacon header looking for beacon type. The APP process only Sm-Covid-19 beacon type and Google Apple GAEN beacon type (BEACON). Every time a BEACON is detected, the routines for estimating the distance and calculating the exposure time are started. At regular intervals, the APP (via its authentication token) sends the following data to the server (Figure 3):

- PID1 issued by APP1
- PID2 issued by APP2
- Duration of the contact (timing)
- Instantaneous and Average distance (over the duration) of the contact
- Detected RSSI
- TxPower (if available)

In an entirely voluntary way, the user can decide to share location data as well. In this case, the server will also receive Latitude, Longitude, Precision, and Provider used data of the APP1. Every APP could send multiple times the same data (generating duplicates) due to upload failures or network issues. The backend only allows writing operations. There is no way for the APP to UPDATE, REMOVE or READ stored data.

*Figure SEQ Figure \* ARABIC 3: Reception of the UUID sent by APP2.*
