# Supplementary material for: Evaluating Epidemiological Risk by Using Open Contact Tracing Data: Correlational Study
Source: J Med Internet Res. 2021 Aug 2;23(8):e28947. doi: 10.2196/28947 (PMC8330631; doi:10.2196/28947)
Supplement: Multimedia Appendix 2 [file jmir_v23i8e28947_app2.docx]

## **Multimedia Appendix 2**

**OpenData Format**

SM-Covid-19 uses a NO-SQL data storage system to ensure scalability and performance. At regular intervals, SM-Covid-19 backend generates a complete dump of the dataset. The dump is converted into a relational database stored into a CSV formatted file to allow the open data to be easy to consult and process. The CSV file is structured as follows:

PID1 and PID2 fields are pre-processed via SHA256 hash with a seed stored into the SoftMining backend system. The CSV is finally cleaned to remove duplicates.

- Date of the contact (dd/MM/YYYY)
- Time of the contact (HH:MM:SS)
- PID1 (256-bit hex)
- PID2 (256-bit hex)
- Contact duration (Integer, in seconds)

Contact distance (Float, in meters)
