# Supplementary material for: Evaluating Epidemiological Risk by Using Open Contact Tracing Data: Correlational Study
Source: J Med Internet Res. 2021 Aug 2;23(8):e28947. doi: 10.2196/28947 (PMC8330631; doi:10.2196/28947)
Supplement: Multimedia Appendix 3 [file jmir_v23i8e28947_app3.docx]

## **Multimedia Appendix 3**

**Dumping and anonymization**

The data stored by the Sm-Covid-19 Backend contains the id of the application instance that generated the data, the proximity ID (which is different from the instance id) of the detected application instance, date and time of the detection, average distance, and duration of the detection. If activated voluntarily by the user, GPS data related to the position of the contact is also available. The data is stored in a Firestone database and is deleted after 21 days.

Before deletion, a data dump is performed, followed by re-anonymizing the data and deleting the GPS data. The raw data acquired by the app are processed to generate the dump as follows:

- A SHA1 function is applied to Instance IDs and Proximity IDs. This function uses a fixed jump maintained in a secure server. This makes it impossible for the server owner to trace the instance ID that generated the data and blurs the detected Proximity ID. Currently, there are no known dictionary attacks or collisions to SHA1. For research reasons, the jump can be requested from the authors.
- The anonymous dump is stored on a secure server and identified as a top-level dump.
- From the top-level dump (re-anonymized full dump), all double lines and spurious lines are deleted, resulting in a second-level dump.
- GPS data, even if provided completely voluntarily by users, is deleted from public open data, thus generating a third-level dump (public dump). Deleting GPS data from open data makes contacts no longer associable with a specific location. The dump containing the GPS data (second level) can be requested from the authors for research purposes only.
