# Supplementary material for: Evaluating Epidemiological Risk by Using Open Contact Tracing Data: Correlational Study
Source: J Med Internet Res. 2021 Aug 2;23(8):e28947. doi: 10.2196/28947 (PMC8330631; doi:10.2196/28947)
Supplement: Multimedia Appendix 4 [file jmir_v23i8e28947_app4.docx]

## **Multimedia Appendix 4**

## **Statistical analysis and estimate of the real number of positives**

As part of our estimate of the possible real infections that may have occurred during the epidemic, we obtained the daily number of the newly performed test using equation 1:

| NT_(k)_ = TT_(k)_ – TT_(k-1)_ | Eq. 1 |
| --- | --- |

Where NT_(k)_ is the number of new tests performed on day k, TT_(k)_ is the cumulative total of tests performed up to day k (included), and TT_(k-1)_ is the cumulative total of tests performed up to day k-1 (i.e., the day before k).

We performed a 7-days sliding window smoothing for both the number of new daily positives and the number of new daily tests. We have calculated the daily ratio between the number of new tests performed and the number of reported new positives using equation 2:

| $r_{(k)} =\frac{{ST}_{(k)}}{{SP}_{(k)}}$ | Eq. 2 |
| --- | --- |

Where ST_(k)_ is the smoothed number of tests performed on day *k,* and SP_(k)_ is the number of reported new positives on day *k*. For each day *k,* we considered the minimum r for ±7 days, therefore using a sliding window of 15 days.

The estimated number of new positives EP_(k)_ for each day is calculated by equation 3:

| ${EP}_{(k)} =\frac{{ST}_{(k)}}{r_{(min\pm7)}}$ | Eq. 3 |
| --- | --- |
