# Supplementary material for: Evaluating Epidemiological Risk by Using Open Contact Tracing Data: Correlational Study
Source: J Med Internet Res. 2021 Aug 2;23(8):e28947. doi: 10.2196/28947 (PMC8330631; doi:10.2196/28947)
Supplement: Multimedia Appendix 5 [file jmir_v23i8e28947_app5.docx]

## **Multimedia Appendix 5**

## **Contact Index and Alpha**

The probability of contagion is calculated considering the number of contacts, duration, and the time spent by the contacts themselves (equation 4). To be effective from an epidemiological point of view, it has been considered that contact must not be fleeting and must take place at a distance of less than one meter. A logistic function was chosen to express the probability of transmission. c_1_ is a coefficient that helps to fine-tune the minimum duration of effective contact for transmitting the virus. The value of parameter c_1_, initially set at 20 minutes^1^ and subsequently calculated more precisely, represents the point of inflection of the function. The time distance of contact is not monotonous but has been mapped using a Gaussian function. The probability of being contagious depends on how many days have passed since contact with an infected person. If contact had just occurred (very recently), the virus would not have had the time to reach a dangerous concentration.

On the other hand, a time of more than ten days without developing symptoms suggests a lower risk of being a carrier. The choice of a Gaussian function, due to its symmetry, would not seem the most suitable, but it has the advantage of very short calculation times, both frontend and backend. The incubation period is defined as the time between infection and onset of symptoms. It is estimated as the time between exposure and report of noticeable symptoms. We used the incubation period distribution calculated by L. Yang *et al*.^2^ The distribution is lognormal with a mean of 5·5 days, a median of 5·2 days, and a standard deviation of 2·1 days. The parameter c_2_ represents the center of the curve, i.e., the average incubation time, and was initially set to 5·1 days.^3^ c_3_ represents the width of the curve, i.e., the time window in which it is reasonable to expect the onset of symptoms after effective contact and was initially set to 11·5 days.^3^ The product of these two functions represents the risk associated with a single effective event (equation 4). It is important to note that equation 1 shows a variation in risk over time since it must be considered that a remote event has no relevance for the onset of symptoms. The coefficient c_0_ is a normalization parameter to have the maximum function equal to 1. The summation takes place on all contacts i.

| $CI=\sum_{i} c_{0}\cdot\frac{e^{\left( t-c_{1} \right)}}{1+e^{\left( t-c_{1} \right)}}\cdot e^{-\frac{\left( \Delta t-c_{2} \right)^{2}}{c_{3}}}$ | Eq. 4 |
| --- | --- |

The simple summation of an indefinite number of risk values associated with contacts would lead to a non-limited value. A threshold value could quickly be introduced so that any person who has had more than a certain number of effective contacts would be considered at risk. We preferred not to use additional arbitrary parameters and calculate the risk factor, as indicated in equation 5. It is a sigmoid starting from its inflection because z is always >0, as linear with slope k and asymptotically to A.

| $\alpha=A\cdot\left( 2\cdot\frac{e^{k\cdot CI}}{e^{k\cdot CI}+1}-\frac{1}{2} \right);0<A<1$ | Eq. 5 |
| --- | --- |

The optimization of these parameters will be the subject of future studies.

**References**

1. Xiao Y, Torok ME. Taking the right measures to control COVID-19. The Lancet Infectious Diseases. 2020;20(5):523-4.

2. Yang L, Dai J, Zhao J, Wang Y, Deng P, Wang J. Estimation of incubation period and serial interval of COVID-19: analysis of 178 cases and 131 transmission chains in Hubei province, China. Epidemiol Infect. 2020;148:e117-e.

3. Lauer SA, Grantz KH, Bi Q, Jones FK, Zheng Q, Meredith HR, et al. The incubation period of coronavirus disease 2019 (CoVID-19) from publicly reported confirmed cases: Estimation and application. Ann Intern Med. 2020;172(9):577-82.
